# A randomised controlled trial of recovery focused CBT for individuals with early bipolar disorder

**DOI:** 10.1186/1471-244X-12-204

**Published:** 2012-11-21

**Authors:** Steven Jones, Lee D Mulligan, Heather Law, Graham Dunn, Mary Welford, Gina Smith, Anthony P Morrison

**Affiliations:** 1 Spectrum Centre for Mental Health Research, Faculty of Health and Medicine, Lancaster University, Lancaster, LA1 4YT, UK; 2 Spectrum Centre for Mental Health Research, School of Health and Medicine, Lancaster University & Greater Manchester West Foundation Trust, Lancaster, UK; 3 Greater Manchester West Foundation Trust, Manchester, UK; 4 School of Medicine, University of Manchester, Manchester, UK; 5 School of Psychological Sciences, University of Manchester, Manchester, UK

## Abstract

**Background:**

There is increasing evidence for the effectiveness of structured psychological therapies for bipolar disorder. To date however there have been no psychological interventions specifically designed for individuals with early bipolar disorder. The primary objective of this trial is to establish the acceptability and feasibility of a new CBT based intervention (Recovery focused CBT; RfCBT) designed in collaboration with individuals with early bipolar disorder intended to improve clinical and personal recovery outcomes.

**Methods and design:**

This article describes a single blind randomised controlled trial to assess the feasibility and acceptability of RfCBT compared with treatment as usual. Participants will be recruited from across the North West of England from specialist mental health services and through primary care and self referral. The primary outcome of the study is the feasibility and acceptability of RfCBT as indicated by recruitment to target and retention to follow-up as well as absence of untoward incidents associated with RfCBT. We also intend to estimate the effect size of the impact of the intervention on recovery and mood outcomes and explore potential process measures (self appraisal, stigma, hope and self esteem).

**Discussion:**

This is the first trial of recovery informed CBT for early bipolar disorder and will therefore be of interest to researchers in this area as well as indicating the wider potential for evaluating approaches to the recovery informed treatment of recent onset severe mental illness in general.

**Trial registration number:**

ISRCTN43062149

## Background

Bipolar disorder (BD) has a prevalence rate of 1.5% [1] affecting over 1 million people in England alone [2]. In addition to repeated periods of mania and depression most individuals with BD experience extended periods of distressing subsyndromal mood symptoms between episodes [3-5]. Consequently BD has significant impact emotionally and functionally [6] and constitutes a substantial financial burden to society: McCrone recently estimated cost to the English economy at £5.2 billion per annum [2].

The National Institute for Clinical Excellence has recommended the provision of structured psychological therapy for individuals with bipolar disorder [7]. To date, controlled trials of structured psychological therapy have focused on individuals with a chronic bipolar disorder. For example, participants in the Lam et al. [8] cognitive behaviour therapy (CBT) trial had experienced a mean of 5.8 depressive and 5.5 manic episodes, whilst in Miklowitz et al.’s [9] comparison of CBT with Family Focussed Therapy and Interpersonal and Social Rhythm Therapy two thirds of the sample had more than 10 episodes of mania and depression at baseline assessment. Although it might seem logical to focus an intervention requiring significant therapist time on individuals with an established illness course recent psychological models of bipolar disorder suggest otherwise. It has been argued by several researchers that associative mechanisms build up over repeated mood episodes in BD such that later episodes are both more readily triggered by psychosocial circumstances previously linked to mood changes and are also less likely to be mediated by cognitive processes [10-12]. This suggests that cognitive-behavioural interventions may be more powerful when applied earlier in the illness course before strong associative links to bipolar emotional states are established. Consistent with this research a recent study of CBT for relapse prevention in bipolar disorder found a significant benefit only for those with fewer episodes in a post hoc analysis [13]. Additionally, there is evidence that individuals with earlier onset of bipolar disorder tend to have worse clinical outcomes, leading to calls for more timely detection and intervention [14,15].

Despite the arguments in favour of earlier treatment there have to date been no RCT evaluations of CBT for bipolar disorder specifically targeted at individuals early in their illness course. The current study will fill this gap employing an adapted CBT intervention for individuals within the first five years since onset of bipolar disorder. An earlier version of this intervention has already proved to be acceptable and feasible in a single case series of 7 bipolar participants [16]. Substantial reductions in subsyndromal symptoms were observed during intervention and six month follow-up along with changes in appraisal styles and stability of sleep/wake cycles. This suggests that psychological intervention for individuals early in their course of bipolar disorder is both effective and acceptable to service users.

The present trial builds on this pilot work in a RCT feasibility study but also extends the intervention to incorporate a clear focus on recovery outcomes (Recovery focused CBT; RfCBT). Recovery in relation to mental health has been defined as “a deeply personal, unique process of changing one’s attitudes, values, feelings, goals, skills and/or roles. It is a way of living a satisfying, hopeful, and contributing life even with limitations caused by the illness. Recovery involves the development of new meaning and purpose in one’s life as one grows beyond the catastrophic effects of mental illness” [17]. Consistent with this individuals with severe mental health issues including bipolar disorder have highlighted the importance of increased hope, sense of control and sense of social connectedness in recovery in contrast with a traditional psychiatric focus on symptomatic outcomes [18-21]. Recovery informed interventions are now encouraged by the UK Government as a mechanism for improving service provision in mental health through personalised care based on individual needs [22,23]. Key elements of this approach include choice, self-determination and self-management [24] through which the client is supported to plan their own route to recovery [23].

Research into recovery and quality of life shows that individuals with bipolar disorder highlight the importance of functional as well as symptom outcomes [21,25]; with a particular focus on engaging or re-engaging in valued activities including employment. Since the very high societal cost of bipolar disorder is driven in large part by loss to the workplace of individuals who could otherwise contribute significantly, supporting individuals in their recovery goals could have clear societal as well as individual benefits. The intervention described in this study was therefore developed based on evidence-based principles for effective psychological interventions for bipolar disorder [26], our pilot case series work and through qualitative interviews, focus group work and consultation with individuals with experience of bipolar disorder.

In this study we evaluate the feasibility and acceptability of delivering RfCBT to individuals with bipolar disorder within 5 years of diagnosis. As a feasibility study we primarily evaluate recruitment into the study and consent to participate, adherence to the intervention, retention within both arms across assessment, intervention and follow-up periods and outcome parameter estimates. From this we will be able to assess the acceptability of the intervention to service users. In addition, the trial will also provide preliminary indications of the impact of RfCBT on self-reported recovery, bipolar relapse, mood symptoms and functioning to inform the selection of primary outcomes for a future definitive RCT.

## Method

This trial is conducted by a multidisciplinary team of researchers, clinicians, statistician and therapists across academic institutions and NHS Trusts in the North West of England. This study was reviewed and approved by the UK NHS Ethics Committee process (REC ref: 10/H1014/60).

### Objective

To determine the feasibility and acceptability of a recovery informed cognitive behaviour therapy intervention (RfCBT) for bipolar disorder compared with treatment as usual.

Main research questions:

● To demonstrate feasibility of recruitment and consenting procedures, adherence to protocol and retention to both arms of the trial across assessment, intervention and follow-up periods.

● To provide parameter estimates of clinical outcomes with respect to, recovery, bipolar relapse, mood, cognitive style, quality of life, functioning, hope, stigma and self esteem, post traumatic growth and medication adherence.

### Trial design

A rater-blind randomised controlled trial which compares: i) Up to 18 h of RfCBT for bipolar disorder with; ii) treatment as usual.

The trial is based in the North West of England with recruitment across this region sampling individuals across rural and urban settings with varied sociodemographic status and ethnic mix.

Randomisation is carried out by the independent Clinical Trials Unit at The Christie NHS Foundation Trust, Manchester, with minimisation on number of bipolar episodes and level of current mood symptoms (including depression and mania). These minimisation variables were selected as there is preliminary evidence that clinical outcomes are better in bipolar disorder for individuals with fewer episodes [13] and with less severe mood problems at inception (although this research is clearest with respect to symptoms of depression the high rates of manic symptoms found in individuals in depressed states indicates the importance of allowing for both affective poles [27,28]).

### Sample size

As the primary purpose of the study is to evaluate the feasibility and acceptability of delivering the proposed intervention a formal power calculation is not appropriate. It has been estimated that 30 participants per group will be sufficient to be able to reliably determine primary feasibility outcomes. The recruitment target is set at 72 participants in total to allow for expected attrition rates and measures of clinical outcome will be recorded at baseline and follow-up to provide an indication of the effectiveness of the intervention in enhancing recovery, regulating mood and its impact on other clinical outcomes. This number will also allow us to evaluate the secondary objective of the trial; to estimate the potential treatment effect size as the basis and justification for a substantive trial.

### Recruitment

Eleven NHS Trusts in the North West UK are taking part in this study (Greater Manchester Mental Health NHS Foundation Trust, Bolton Primary Care Trust, Salford Primary Care Trust, Trafford Primary Care Trust, Manchester Mental Health and Social Care Trust, Manchester Primary Care Trust, Lancashire Care NHS Foundation Trust, North Lancashire Primary Care Trust, 5 Boroughs Partnership NHS Foundation Trust, Cumbria Partnership NHS Foundation, Cumbria Primary Care Trust). Community mental health teams, out-patient clinics, GP surgeries, primary care mental health teams and voluntary services are approached to identify potential participants. Care co-ordinators, research nurses and research development officers are also approached in order to contact potential participants in the first instance. When recruiting in community mental health teams and voluntary services, a member of the research team presents an outline of the study and provides written material about the study. Potential participants are offered a participant information sheet by their care co-ordinators or the research team, outlining the study and their role should they wish to take part.

The study is also advertised in local media and posters and leaflets distributed in both NHS and non NHS sites to maximise participant access. Care co-ordinators and other relevant health professionals are informed of a participant’s involvement in the study subject to participant consented. If a participant does not wish for their GP or care coordinator to be informed about their involvement in the study, this does not prevent their participation and contact details of all health professionals involved in their care are still taken in case of any clinical adverse events during the study. Participants are made aware on entry to the study that their care co-ordinator will be contacted should they be a significant risk to themselves or others during the study.

### Inclusion/exclusion criteria

Potential participants must meet the inclusion criteria of:

● DSM-IV diagnosis of primary bipolar disorder with onset in last 5 years [29].

● Sufficient understanding of written and spoken English in order to provide consent, engage with interviews and use the intervention.

● Aged between 18–65 years.

Exclusion criteria:

● Manic, hypomanic, depressed or mixed episode currently or in the last four weeks

### Outcome measures

In order to evaluate the feasibility and acceptability of delivering the RfCBT intervention to individuals with a recent diagnosis of bipolar disorder (onset in last 5 years) the following data will be evaluated: levels of recruitment into the trial; retention of participants in both arms of the study and adherence to and completion of the intervention. At baseline the Structured Clinical Interview for DSM-IV is completed to confirm bipolar diagnosis and to provide information on comorbid disorder diagnoses as well as information on sociodemographic variables. Measures of clinical outcome will be recorded at baseline and follow-ups to provide an indication of the effectiveness of the intervention in increasing time to bipolar relapse, self reported recovery score and improving observer rated mood as primary clinical outcomes. Secondary outcomes are levels of self reported affective symptoms, functioning/quality of life and medication adherence. Process measures are depressive and hypomanic appraisal style, hope, stigma, self-esteem and post trauma growth.

### Primary clinical outcomes

Hypotheses for the primary clinical outcomes are that RfCBT will: i) increase self reported recovery as measured by the Bipolar Recovery Questionnaire [30]; ii) increase time to bipolar relapse measured by the Structured Clinical Interview for Diagnosis: Research Version (SCID DSM-IV: SCID Life [31]; iii) reduce mood symptoms as measured by Hamilton Depression Rating Scale (HAM-D) [32] and Bech-Rafaelsen Mania Scale (MAS) [33].

### Measurement of secondary outcomes

Hypotheses for secondary outcomes are that RfCBT will improve; i) self reported mood symptoms as measured by the Beck Depression Inventory (Second Edition) (BDI-II)[34] and Internal States Scale (ISS) [35]; ii) Quality of life and social functioning as measured by Personal and Social Functioning Scale [36] and the Brief Quality of Life in Bipolar Disorder Questionnaire. (QoL.BD) [25]; iii) Medication adherence measured by the Stephenson Medical Adherence Questionnaire (MEDAD) [37].

### Process measures

It is hypothesised that RfCBT will improve clinical outcomes through; i) reducing tendency towards positive self appraisals as measured by the Hypomanic Interpretations Questionnaire and Interpretations of Depression Questionnaire(HIQ/IDQ) [38,39]; ii) reducing experiences of stigma measured by the Hayward Stigma Questionnaire-*Revised Version* (HSQ) [40]; *iii*) increasing hope and self-esteem as measured by the Herth Hope Index [41] and the Self-Esteem Rating Scale (SERS) [42]; iv) increasing post trauma growth as measured by the post trauma growth inventory [43].

### Measures to assess therapeutic alliance and adherence to treatment protocol

Engagement in therapy will be assessed by means of the Work Alliance Inventory (short-form, therapist and client versions; WAI-S) [44]. Treatment fidelity will be assessed by both the Cognitive Therapy Scale Revised version (CTS-R) [45] and the RfCBT Fidelity Scale specifically designed for the current study.

### Schedule of assessments

**Figure 1:**
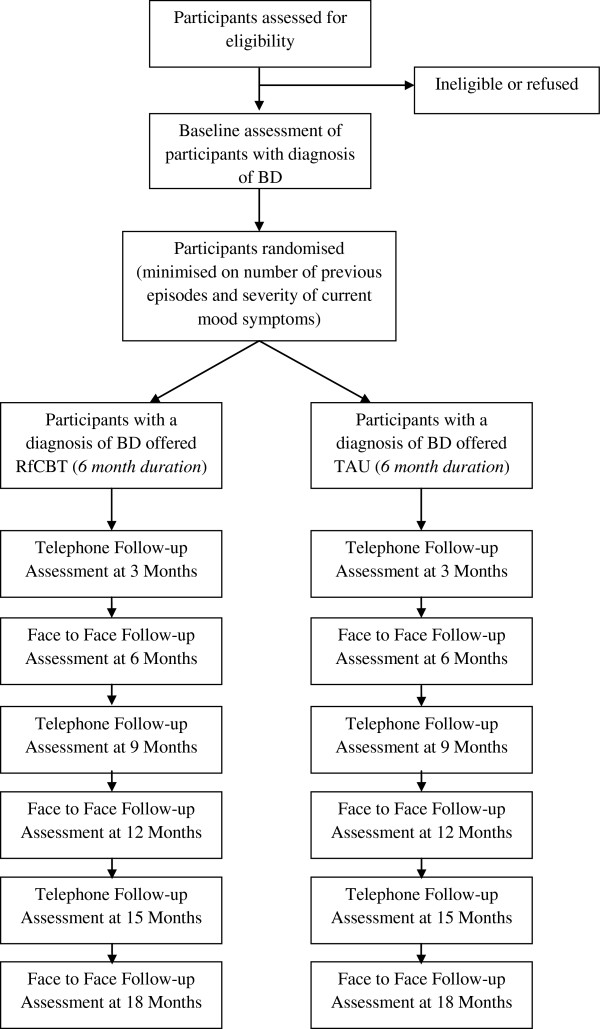
CONSORT Diagram showing Design of the Study.

The follow-up period will be up to 18 months from initial randomisation. In addition to regular (3 monthly) assessments to evaluate bipolar relapse, recovery and observer rated mood: additional clinical outcome and process measures will be assessed at inception and then 6 monthly. See Figure 1 (CONSORT diagram) and Table 1 for more details on the timing and type of specific assessments.

**Table 1:** Schedule of Quantitative Assessments for Service Users

**Assessment**	**0 weeks(baseline)**	**3 Months(t)**	**6 Months**	**9 Months(t)**	**12 Months**	**15 Months(t)**	**18 Months**
***Primary Outcome Measures***							
Bipolar Recovery Questionnaire (BRQ)	*	-	*	-	*	-	*
Time to bipolar relapse: weekly LIFE scores (SCID life)	*	*	*	*	*	*	*
Hamilton Depression Rating Scale (HDRS)	*	*	*	*	*	*	*
Bech-Refaelsen Mania Scale (MAS)	*	*	*	*	*	*	*
***Secondary Outcome Measures***							
Beck Depression Inventory (BDI)	*	-	*	-	*	-	*
Internal States Scale (ISS)	*	-	*	-	*	-	*
Brief Quality of Life in Bipolar Disorder Questionnaire (QoL.BD)	*	-	*	-	*	-	*
Personal and Social Performance Scale (PSP)	*	-	*	-	*	-	*
Stephenson Medication Adherence Interview (SMAI)	*	-	*	-	*	-	*
***Process Measures***							
Hypomanic Interpretations Questionnaire (HIQ)	*	-	*	-	*	-	*
Interpretation of Depression Questionnaire (IDQ)	*	-	*	-	*	-	*
Hayward Stigma Questionnaire – *Revised Version* (HSQ)	*	-	*	-	*	-	*
Self Esteem Rating Scale (SERS)	*	-	*	-	*	-	*
Herth Hope Inventory (HHI)	*	-	*	-	*	-	*
Post Trauma Growth Inventory (PTGI)	*	-	*	-	*	-	*

* Measure used at assessment.

- Measure not used at assessment.

(t) Telephone assessment.

### Individual recovery focused CBT intervention

The intervention is informed by current evidence for components of effective CBT interventions which include mood monitoring and awareness, regularisation of routines, enhancing prodromal coping and problem solving training [26,46]. Prior to this study we also completed a case series of CBT for first diagnosis bipolar clients (although this had a less specific recovery focus), which indicated the importance of maintaining a very flexible engagement approach, allowing time for full consideration of meaning of diagnosis to the client, engaging in coping skills reviews around subsyndromal exacerbations as well as prior episodes and considering the role of appraisals of fluctuations in affect. These lessons have also informed the final treatment manual. In addition we have conducted qualitative interviews with individuals about their experiences of recovery in bipolar disorder [21] and focus groups with individuals with bipolar disorder to consult on the draft content, format and supporting materials for the RfCBT intervention. The intervention is delivered by mental health professionals trained to BABCP accreditation level in CBT or equivalent. Duration of therapy is up to 18 h delivered over a period of approximately six months at client’s homes or mental health facilities according to client preference. Initial sessions are weekly, with later sessions fortnightly. Sessions are typically 45–60 min long.

The following elements are contained within the manual and reflect elements typically addressed in the course of therapy although the relative emphasis on each element is informed by the client’s formulation:

● Introducing the recovery approach to clients

● Collection of information about current and historical mood and functioning

● Meaning and relevance of diagnosis

● Identification of recovery informed therapy goals

● Initial formulation of relationships between mood experiences and progress towards recovery goals

● Identification and application of CBT techniques to address and facilitate positive coping

● Consideration of wider functioning issues in relation to recovery

● Development and completion of recovery plan

● Sharing lessons from therapy with key stakeholders

The therapy approach differs from standard CBT for bipolar disorder in the following ways:

– Explicit focus on eliciting client focussed goals rather than presuming a target of relapse prevention.

– Formulation driven idiosyncratic approach rather than applying a very similar model of bipolar experience across clients.

– Freedom to work within whatever model the client brings.

– Openness to address functioning and comorbidity issues as well as mood problems.

### Analysis

#### Feasibility

As the primary purpose of the study is to evaluate the feasibility and acceptability of delivering the proposed intervention a formal power calculation comparing treatment groups is not essential. With 72 subjects in total the study will estimate a follow-up rate of 75% with precision +/−10%.

#### Clinical outcomes

All therapy effects will first be estimated using the intention-to-treat principle, supplemented by estimation of the therapy effects in those participants who actually receive the intervention via estimation of the Complier-Average Causal effect or CACE [47,48]. Time to relapse will be assessed by survival analysis (using either proportional hazard or accelerated life-time models). Each of the secondary outcome measures will be analysed with analysis of covariance through the use of generalised linear models (the data distribution and link function being dependent upon the outcome under consideration). Adjustments for missing data will be made assuming that the missing data mechanism is either ignorable or latently ignorable [49].

## Discussion

This study will provide important data for the development and evaluation of a future definitive recovery focussed CBT trial for early bipolar disorder. The recovery intervention that is the subject of this study has been developed in partnership with individuals with lived experience of bipolar consistent with Mental Health Research Network good practice guidelines [50] including service user involvement in qualitative work on recovery experiences, development of a recovery outcome measure and structure and format of the RfCBT intervention. These meetings were in addition to the focus group work referred to earlier. This level of engagement of individuals with personal experience of bipolar disorder fits with the model of recovery approaches as being empowering, individualised and grounded in the individual’s own priorities and needs. In addition to this important element, the intervention is also structured around evidence based components of effective psychological therapy including developing strategies for early warning signs of depression and mania and stabilisation of activity and sleep where this meets clients’ needs. It differs from standard CBT in that there is an explicit focus on eliciting client goals which may not include relapse prevention as primary, where in CBT this target is assumed for all clients. There is also a strong emphasis on formulation rather than applying a very similar model of bipolar experience across clients, so the client route through therapy is different depending on their needs, giving the therapist freedom to work with whatever model the client brings but in the context of offering evidence based approaches. Therapists are also explicitly permitted to focus on issues around functioning and comorbidity as well as mood problems.

Strengths of the study include targeting a clearly defined sample who are currently poorly served by available interventions, namely individuals with recent onset bipolar disorder. It is also the first therapy for bipolar disorder which explicitly aims to enhance recovery outcomes which are valued by service users and supported by government policy. Additionally, we have not assumed expert clinician knowledge of the nature of recovery in bipolar disorder, but rather grounded the intervention in the experience and knowledge that service users have offered us with respect to recovery. The study is also recruiting from across NHS primary and secondary care settings and through self referral so that findings should be more representative than those that solely focus on specialist mental health settings as only a subset of individuals with bipolar disorder are in such settings long term [51].

There are weaknesses to the study which would need to be addressed in a definitive trial. Firstly, there is no active treatment control group so that any indications of effectiveness need to be interpreted with caution as we will not know whether possible benefits are a function of this specific treatment or structured treatment in general. Secondly, the scale of the study allows us to follow participants for up to 18 months following therapy completion. Longer follow-ups would be helpful to indicate more definitively whether this intervention impacts on relapse as well as recovery. Thirdly, the very individualised nature of the intervention means that it was necessary to devise a new measure specifically to explore fidelity to a very individualised therapy protocol. As we are uncertain how this will perform we have also used the established CTS-R which might underestimate fidelity in a flexible therapy of this type.

Despite these challenges, if the current study indicates that RfCBT is feasible and has potential clinical benefits it will be an important step towards developing evidence-based recovery interventions for people with bipolar disorder that have been lacking until now.

## Competing interests

The authors declare that they have no competing interests.

## Authors' contributions

SJ is the Principal Investigator for the current study responsible for the conduct of the study and wrote the paper. LM contributed to recruitment and follow-up of study participants and contributed to protocol design and write up of the current paper. HL co-ordinated the study in the context of the wider Recovery Programme. GM is the trial statistician and is a grant holder. MW has clinical oversight of the recovery programme. GS contributed to therapy protocol development. TM is Chief Investigator for the Recovery Programme as a whole. All authors contributed to the design of the study, revised the manuscript and gave final approval to the manuscript.

## Pre-publication history

The pre-publication history for this paper can be accessed here:

http://www.biomedcentral.com/1471-244X/12/204/prepub
